# Exenatide Attenuates Non-Alcoholic Steatohepatitis by Inhibiting the Pyroptosis Signaling Pathway

**DOI:** 10.3389/fendo.2021.663039

**Published:** 2021-04-19

**Authors:** Yu Liu, Da-Wei Wang, Dan Wang, Bin-Hong Duan, Hong-Yu Kuang

**Affiliations:** ^1^ Department of Endocrinology, The First Affiliated Hospital of Harbin Medical University, Harbin, China; ^2^ Department of Endocrinology, Heilongjiang Provincial Hospital, Harbin, China; ^3^ Department of General surgery, The First Affiliated Hospital of Harbin Medical University, Harbin, China

**Keywords:** exenatide, pyroptosis, gasdermin D, non-alcoholic steatohepatitis, therapeutic target

## Abstract

**Background/Aims:**

Exenatide is a glucagon-like polypeptide-1 analog, whose main clinical use is to treat type 2 diabetes. However, the mechanism of exenatide in mitigating non-alcoholic steatohepatitis (NASH) remains unclear. This study aimed to investigate the *in vitro* and *in vivo* effect of exenatide on NASH.

**Methods:**

Leptin receptor-deficient C57BL/KsJ- db/db male mice were fed with methionine-choline-deficient (MCD) diet for 4 weeks to induce NASH, while oleic acid/LPS-treated HepG2 cells were used as an *in vitro* cell model. Exenatide (20 µg/kg/day, subcutaneous) and specific exenatide inhibitors (20 µg/kg/day, intraperitoneal) were used to determine the effects of exenatide on NASH.

**Results:**

Exenatide treatment inhibited the pyroptosis signaling pathway to attenuate NASH.

**Conclusion:**

To the best of our knowledge, this report provides the first evidence showing that exenatide attenuated NASH by inhibiting the pyroptosis signaling pathway. Exenatide thus has important pathophysiological functions in NASH and may represent a useful new therapeutic target.

## Introduction

Exenatide is a glucagon-like polypeptide-1 (GLP-1) analog initially discovered in saliva of the American tailed poisonous lizard. The molecular structure of exenatide is homologous to GLP-1, and it binds to GLP-1 receptors in humans and acts like GLP-1. Exenatide has been shown to effectively control blood glucose, improve islet function, reduce body weight, and to have a cardioprotective effect (1–3). The use of exenatide for the treatment of type 2 diabetes also has a cardiovascular protective effect, by reducing systolic blood pressure and the incidences of heart failure, myocardial ischemia, and myocardial infarction (4, 5). However, the role of exenatide in the clinical treatment of non-alcoholic steatohepatitis (NASH) is unclear, and further studies are needed to provide a theoretical basis for the clinical application.

Non-alcoholic fatty liver disease (NAFLD) is a disease with a high worldwide incidence. Improvements in domestic living standards, living habits, and dietary structure have been accompanied by increases in the incidences of both obesity and diabetes, while the incidence of NAFLD has also increased to become the second most common liver disease after viral hepatitis (6). NAFLD mainly comprises simple fatty liver, steatohepatitis, and cirrhosis, of which 15–25% of cases develop NASH (7, 8). The pathology of NASH involves the accumulation of lipid droplets with evidence of cell damage, inflammation, and hepatic fibrosis (8), with 3–15% of patients with NASH progressing to cirrhosis with hypertension, liver failure, hepatocellular carcinoma, and other complications (7, 9). NASH is currently considered to be a necessary stage in the development of liver cirrhosis and an inflection point for the deterioration of NAFLD, and it is the most rapidly growing cause of hepatocellular carcinoma among US patients listed for liver transplantation (10). Other than lifestyle modification through diet and exercise, there are currently no other approved treatments for NASH/NAFLD. Although weight loss can be effective, it is difficult to achieve and sustain (11). The aim of NASH therapy is to reduce patient mortality, and to minimize the occurrence and development of liver and cardiovascular complications, clinical symptoms, and improve the patient’s quality of life. However, the mechanism of liver damage and disease in NASH remains unclear. The current study therefore aimed to characterize the pathogenetic mechanisms and potential new therapeutic targets for NASH.

Pyroptosis is a type of inflammatory programmed cell death that depends on the activity of caspase-1 or caspase-11 (12). Activation of the inflammasome is a key process in cell death. Studies have shown that, under various disease conditions, nucleotide-binding oligomerization domain-like receptor protein 3 (NLRP3) recognizes non-microbial danger signals and mediates cell death in non-bacterial inflammatory diseases such as type 2 diabetes, *Helicobacter pylori* gastritis, and cardiovascular disease (13, 14). Cell death has also been reported to be mediated by gasdermin D (GSDMD) protein, whose N-terminus migrates to the cell membrane to form pores, promote the release of inflammatory factors, and mediate the occurrence of pyroptosis (15, 16). However, the effect of exenatide on pyroptosis is still unknown.

This study therefore aimed to determine the ability of exenatide to inhibit pyroptosis in non-alcoholic steatohepatitis. A deeper understanding of NASH will help to identify potential new drug targets for its clinical prevention and treatment.

## Materials and Methods

### Animals

Two-month-old C57BL/KsJ- db/db male mice were purchased from Charles River Laboratories and randomly divided into 4 groups with 6 mice in each group. Mice in the control group were fed with standard chow diet. The mice in other groups were fed with MCD diet (Research Diets, A02082002BR) for 4 weeks to induce NASH. For exenatide treatment group, the mice were injected subcutaneously with exenatide (Baxter Pharmaceutical Solutions, Deerfield, IL, USA) at a dose of 20 µg/kg/day subcutaneously from the beginning of week 2 to the end of week 4. For exendin 9-39 treatment group (exenatide + exendin 9-39), the mice were further injected intraperitoneally with exendin 9-39 (Sigma-Aldrich, St. Louis, MO, USA) at a dose of 20 µg/kg/day (17) on the basis of exenatide administration. Mice in the control and NASH group were administrated with normal saline instead. After 4 weeks, blood samples were collected by retro-orbital bleeding, and the serum was collected after centrifugation (3,000 rpm, 10 min). Then the animals were sacrificed *via* CO_2_ inhalation for the collection of liver samples. The animal study was reviewed and approved by The First Affiliated Hospital of Harbin Medical University (Approval Number: 2019/02/213).

### Cell Culture

HepG2 cells were seeded onto six-well plates at the density of 2×10^6^ cells/well. High glucose Dulbecco’s Minimal Essential Medium (DMEM) containing 10% fetal bovine serum was used for cell culture. Oleic acid was dissolved in DMSO solution. Cells in the Oleic acid/LPS group were first incubated with 200 μM oleic acid for 24 h and then with oleic acid and 2 μg/ml LPS for 12 h (18). Drug-treated cells were simultaneously incubated with oleic acid and exenatide (20 nM) (19), or exenatide (20 nM) plus exendin 9-39 (20 nM) for 24 h, and subsequently supplemented with LPS for 12 h. DMSO was used as the treatment control.

### Electron Microscopy

Liver tissues from each group were cut into 1 cm^3^ pieces and fixed in glutaraldehyde for 24 h. The samples were cut and viewed using an electron microscope (×5,000) to observe liver cell death and the number of inflammatory cells. The average values were then calculated.

### Transaminases and Lipid Metabolism in Serum, HepG2 Cells, and Liver Tissues

Tissue samples were homogenized mechanically in an ice water bath, centrifuged at 2,500 rpm for 10 min, and the supernatant was saved. Total cholesterol (T-CHO) was quantified colorimetrically using cholesterol/cholesteryl ester assay kit (abcam, ab65359). Alanine aminotransferase (ALT) was measured using microplate reader based on the Alanine transaminase activity assay kit (abcam, ab105134). Aspartate aminotransferase (AST) levels were measured using the aspartate aminotransferase activity assay kit (abcam, ab105135). Tissue and serum were treated in a similar manner. Cell suspensions were prepared and centrifuged at 1,000 rpm for 10 min, the supernatant was discarded, and the cells were left to settle. The cells were then lysed in Triton X-100 (1%–2%) for 30–40 min, and the liquid was used immediately after lysis. The triglyceride (TG) content of the samples was determined using a TG assay kit (abcam, ab65336).

### Hematoxylin and Eosin (HE), Masson’s Trichrome and Sirius Red Staining

Liver tissues were dehydrated and fixed in 4% paraformaldehyde, embedded in paraffin, cut into 5 μm thick sections, deparaffinized, and stained with HE (Solarbio, G1120), Masson’s trichrome (Solarbio, G1340) and Picro Sirius Red Stain Kit (Solarbio, G1470). The histology was then determined using a light microscope.

### Quantitive Real-Time PCR

Total RNA was isolated using TRIzol and cDNA was synthesized using the EasyScript first strand cDNA synthesis and gDNA removal kit (Transgen). Relative quantification of gene expression was performed using the 2^-ΔΔCT^ method and GAPDH normalization. Results were expressed as fold changes over values obtained from control. Primers were listed as following: collagen I forward TAAAGGGTCATCGTGGCTTC, reverse ACCGTTGAGTCCATCTTTGC; collagen III forward GCCTTCTACACCTGCTCCTG, reverse CCTCCGACTCCAGACTTGAC.

### Western Blotting

Total protein samples were extracted from liver tissues and HepG2 cells, loaded on 12% sodium dodecyl sulfate-polyacrylamide gels (Beyotime, Biotechnology, Jiangsu, China), and then transferred to polyvinylidene difluoride membranes (BosterBiological Technology, Wuhan, China). The membranes were incubated with primary antibodies toNLRP3 (Cell Signaling Technology, Boston, MA, USA; #15101), caspase-1 (Cell Signaling Technology; #2225), interleukin (IL)-1β (Cell Signaling Technology; #12507), and glyceraldehyde 3-phosphate dehydrogenase (GAPDH) (Cell Signaling Technology; #5174) (1:1000) at 4°C overnight, followed by goat anti-mouse IgG-HRP (Transgen, HS201-01, 1:5000) or anti-rabbit IgG-HRP (Transgen, HS101-1, 1:5000) for 1 h. GAPDH was used as an internal control. The protein bands were measured and analyzed using Quantity One software. Six replicates per group were used for each western blot and two to three independent experiments were performed.

### Oil Red O Staining

Liver tissues of mice in each treatment group were sliced after freezing, then stained with Oil Red O for 15 min. The slides were then washed and counterstained with hematoxylin for 3 min, washed in running water until they turned blue, sealed with glycerol, and observed using a light microscope.

### Immunofluorescence Staining

HepG2 cells were fixed in 4% buffered paraformaldehyde in phosphate-buffered saline for 15 min at room temperature, followed by blocking in 5% BSA for 30 min at 37°. The cells were then treated with primary antibodies against NLRP3 (ab4204), GSDMD (abcam, ab219800, 1:500), caspase-1 (Invitrogen, PA5-17570, 1:500), and IL-1β (Invitrogen, P420B, 1:200) at 4°C overnight, and then incubated with goat anti-rabbit IgG (FITC conjugated, 1:500) and goat anti-mouse IgG (PE conjugated, 1:500) secondary antibody for 1 h at 37°. The nuclei were stained with 4′,6-diamidino-2-phenylindole (Beyotime, Shanghai, China) for 20 min. The images were then captured by fluorescence microscopy (Nikon 80i; Nikon, Tokyo, Japan).

### Immunohistochemical Analysis

Liver samples were fixed with 4% paraformaldehyde, embedded in paraffin, cut into 5 µm thick sections, deparaffinized, and then immunostained with primary antibodies against NLRP3, GSDMD, caspase-1, or IL-1β (1:200) at 4°C overnight, followed by secondary antibody. The sections were then stained with diaminobenzidine and examined by light microscopy. The semiquantitative analysis of Immunohistochemical staining data was performed using the Immunohistochemistry Image Analysis Toolbox in the ImageJ software. Color selection is used to select and reserve the positive color pixels while the background color pixels are eliminated.

### Statistical Analysis

Data were presented as the mean ± standard error of the mean (SEM). Comparisons among multiple groups were analyzed using one-way ANOVA and *post hoc* analysis adjusted for Bonferroni’s multiple comparisons test. Comparisons between two groups were done using an unpaired Student’s t-test. All statistical analyses were carried out using SPSS statistical software for Windows, version 20.0 (SPSS, Chicago, IL, USA) and the graphs were plotted using Prism 5.0 software (GraphPad, La Jolla, CA, USA). P < 0.05 was considered statistically significant.

## Results

### Exenatide Attenuated NASH by Inhibiting the Pyroptosis Pathway *In Vitro*

**Figure 1:**
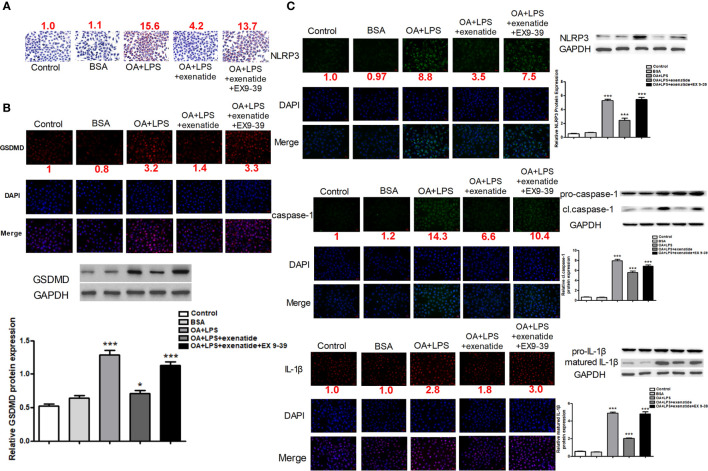
Exenatide attenuated NASH by inhibiting the pyroptosis signaling pathway *in vitro*. **(A)** A representative Oil Red O staining image showed that exenatide attenuated NASH. The numbers above each picture (shown in red) indicated the relative staining intensity as quantified by ImageJ. **(B)** Representative immunofluorescence images of GSDMD and GSDMD protein expression levels measured by western blotting. **(C)** Representative immunofluorescence images of NLRP3, caspase-1, and IL-1β. NLRP3, caspase-1, and IL-1β. Protein expression levels were measured by western blotting. For panel **(B, C)**, the numbers below the images (shown in red) indicated the relative fluorescence intensity as quantified by ImageJ. Two subcultures were used for each western blot markers and the experiment was repeated 2-3 times. *p < 0.1; ***p < 0.001. Data are expressed as the mean ± SEM.

We established invitro NASH cell culture model using Oleic acid/LPS treatment in HepG2 cells, and examined the effect of exenatide on pyroptosis. HepG2 cells were treated with oleic acid + LPS, oleic acid + LPS + exenatide, oleic acid +LPS + exenatide+ exenatide inhibitor (exendin 9-39) and then stained by Oil Red O. The results indicated that exenatide attenuated oleic acid-induced NASH (**Figure 1A**). Representative immunofluorescence images confirmed that exenatide relieved NASH by inhibiting pyroptosis. GSDMD protein expression levels were increased by oleic acid, and this increase was significantly inhibited by exenatide, as demonstrated by western blotting (**Figure 1B**). NLRP3, caspase-1, and IL-1β expression levels increased after treatment with oleic acid to induce NASH, and this increase was significantly reduced by exenatide (**Figure 1C**).

### Exenatide Attenuated NASH in a Mouse Model *In Vivo*

**Figure 2:**
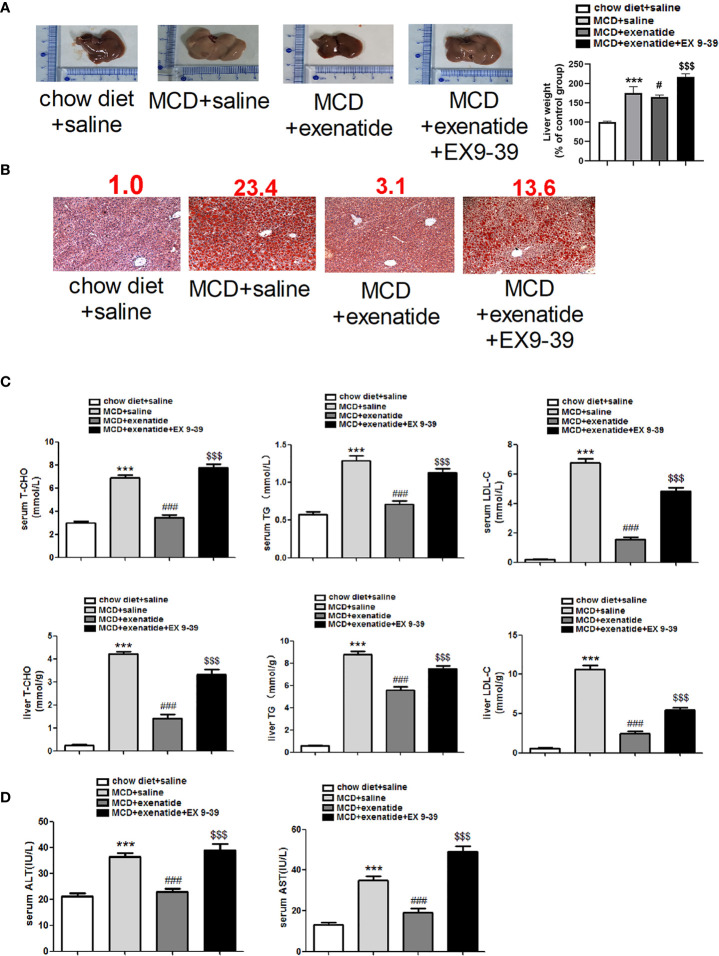
Exenatide attenuated NASH *in vivo*. **(A)** Representative images of liver tissue and the semiquantitative analysis of liver weight. **(B)** Representative Oil Red O staining image. The numbers above each picture (shown in red) indicated the relative staining intensity as quantified by ImageJ. **(C)** Liver tissue and serum levels of T-CHO, TG, and LDL-C were measured to confirm the protective effect of exenatide in NASH. **(D)** The levels of serum ALT and AST. ^***^p < 0.001 vs. chow diet + saline; ^###^p < 0.001 vs. MCD + saline; ^$$$^p < 0.001 vs. MCD + exenatide (n = 5–6). Data are expressed as the mean ± SEM.

Next, we established *in vivo* NASH model using MCD diet induction in db/db mice. Representative light microscope images showed that mice fed with MCD diet had significantly more fat than those fed a normal diet. Mice in the MCD group were also heavier than those in the control group, and this increase in weight was attenuated by exenatide (**Figure 2A**). Histopathological evidence also showed that the MCD diet induced a fatty liver, which was attenuated by exenatide. Liver weight was measured to study the effect of exenatide and to obtain the ratio of body weight/liver (**Figure 2B**). Oil Red O staining of liver tissue confirmed that the MCD diet-induced NASH was attenuated by exenatide (**Figure 2C**). Measurements of T-CHO, TG, and LDL-C in liver tissue and serum all indicated that NASH was attenuated by exenatide (**Figure 2D**). Furthermore, ALT and AST levels in the liver and serum were increased in the NASH model mice, but reduced by exenatide.

### Exenatide Inhibited Fibrosis in the NASH Mouse Model *In Vivo*

**Figure 3:**
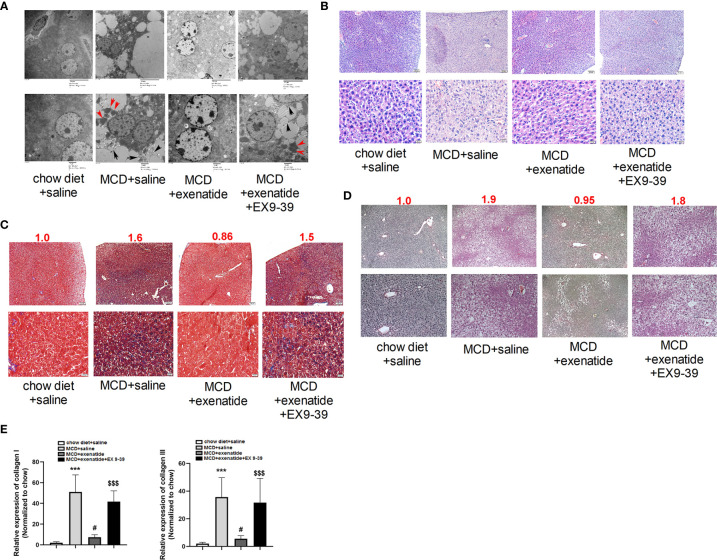
Exenatide inhibited fibrosis in a NASH mouse model. **(A)** Representative electron microscopy images showing pyroptosis bodies (red arrows) and membrane rupture (black arrows) in NASH mice liver tissue and exenatide inhibitor treated mice. Exenatide treatment reversed the phenotypes. **(B)** Representative liver tissue images stained by hematoxylin and eosin. The pathological features of NASH were observed in MCD and MCD+Exenatide+Ex9-39 groups, such as nuclear rupture, increased cell area, and disordered arrangement. **(C)** Representative liver tissue images after Masson staining. The numbers above each picture (shown in red) indicated the relative staining intensity as quantified by ImageJ. **(D)** Representative liver tissue images stained by Sirius Red. **(E)** RT-qPCR analysis of the expression of collagen I and collagen III in mice liver tissues. ^***^p < 0.001 vs. chow diet + saline; ^###^p < 0.001 vs. MCD + saline; ^$$$^p < 0.001 vs. MCD + exenatide (n = 5–6). Data are expressed as the mean ± SEM.

Liver cells from NASH mice were disordered, with many pyroptosis bodies and membrane rupture observed by a transmission electron microscopy, and these effects were reversed by exenatide (**Figure 3A**). HE staining revealed nuclear rupture, increased cell area, and disordered arrangement in NASH mice, while this typical NASH pathological process (20) was reversed by exenatide (**Figure 3B**). Masson and Sirius Red staining showed darker staining of liver tissues in NASH mice and exenatide+Ex 9-39 treated mice, while exenatide treatment reversed the phenotype (**Figures 3C, D**). qPCR analysis of the expression of collagen I and collagen III confirmed the upregulation of collagen synthesis in the NASH mice and exenatide+Ex 9-39 treated mice (**Figure 3E**).

### Exenatide Attenuated NASH in a Mouse Model *via* the Pyroptosis Signaling Pathway

**Figure 4:**
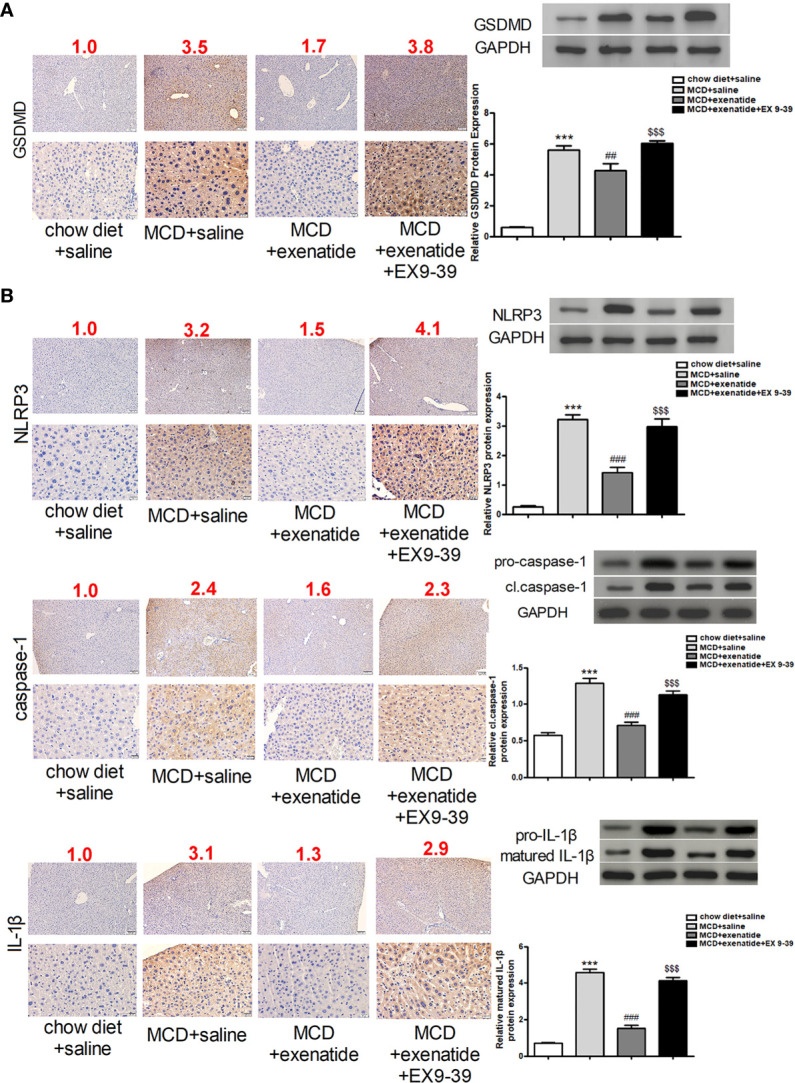
Inhibition of the pyroptosis signaling pathway by exenatide in NASH. **(A)** Representative immunohistochemistry image of GSDMD. Protein expression levels of GSDMD in HepG2 cells as determined by western blotting. **(B)** Representative immunohistochemistry image of NLRP3, caspase-1, and IL-1β. NLRP3, caspase-1, and IL-1β protein expression levels were detected by western blotting. The numbers above each picture (shown in red) indicated the relative staining intensity as quantified by ImageJ. ^***^p < 0.001 vs. chow diet + saline; ^###^p < 0.001 vs. MCD + saline; ^$$$^p < 0.001 vs. MCD + exenatide (n = 6). Data are expressed as the mean ± SEM.

To further understand the effect of exenatide, we examined its underlying mechanism in the MCD diet-induced NASH mouse model. The expression of pyroptosis pathway related proteins were analyzed in mice liver by western blot and immunochemical staining. The results showed that GSDMD protein expression was increased in NASH mice, as confirmed by western blotting (**Figure 4A**). Furthermore, expression levels of the pyroptosis-related factors NLRP3, caspase-1, and IL-1β indicated that the pyroptosis signaling pathway was activated in NASH, and that this effect was attenuated by exenatide (**Figure 4B**).

## Discussion

NAFLD is the most common liver disease worldwide and its incidence is increasing, with NASH accounting for 15%–25% of cases. Importantly, NASH is an important contributory factor to liver cirrhosis and liver cancer, and the available treatments remain inadequate (10). The main pathogenic factors of NASH include obesity, type 2 diabetes, and hyperlipidemia (11). Exenatide is usually used as adjunctive therapy to improve glycemic control in patients with type 2 diabetes mellitus (T2DM) (21). It improves glycemic control through a combination of mechanisms, including glucose-dependent insulin secretion, regulation of glucagon secretion, delaying gastric emptying, and decreasing food intake (22). Previous studies have shown that exenatide could ameliorate mitochondrial TCA cycle flux and significantly decreases steatosis and hepatocyte lipotoxicity in diet induced NASH in mice (23). However, the underlying mechanisms were unclear. The current results demonstrated that exenatide attenuated NASH induced by oleic acid or the MCD diet through inhibiting the pyroptosis signaling pathway, suggesting that exenatide might represent a new therapeutic target for NASH.

Pyroptosis is a type of proinflammatory programmed cell death induced by caspase-1 (24, 25). The NLRP3 inflammasome is activated in response to danger signals and recruits pro-caspase-1. The auto-cleaving of pro-caspase-1 generates the active caspase-1 tetramer (26). Caspase-1 activation in turn induces the secretion of certain proinflammatory cytokines, such as IL-1β, further promoting inflammation. Previous studies have shown that caspase-1-mediated pyroptosis is caused by the executive protein, GSDMD, which promotes the formation of pores in the cell membrane and releases inflammatory factors to mediate pyroptosis (27, 28).

In the current study, we examined the pyroptosis signaling pathway as a target of exenatide by examining the expression levels of the pyroptosis-related factors, NLRP3, caspase-1, GSDMD, and IL-1β in oleic acid- and MCD diet-induced *in vitro* and *in vivo* models of NASH, respectively. We demonstrated that the pyroptosis signaling pathway was activated in NASH. We also used simultaneous administration of exenatide and the exenatide inhibitor, exendin, to clarify the role of exenatide in alleviating NASH by inhibiting the pyroptosis signaling pathway, and showed that exenatide reduced the expression levels of the pyroptosis-related factors. In addition, HE and Masson staining showed that exenatide reduced liver fibrosis and inflammation in NASH. Treatment with exenatide thus relieved oleic acid- or MCD-induced NASH.

Although promising effects of exenatide were observed, our study has certain limitation due to the experimental design. Exenatide were given a week after the MCD diet feeding in all treated animals, rather than at the onset of NASH manifestations which may be barriers to clinical translation. Lack of randomization in mice housing may lead to potential sources of bias. Moreover, the effects were not explored in other NASH models. In summary, we found that exenatide inhibited pyroptosis, and significantly alleviated NASH in our HepG2 cells and mice model, respectively. These findings provided deeper understanding of the pathological mechanisms of NASH, and identified a potential new therapeutic target that could improve the quality of life in patients with NASH.

## Data Availability Statement

The original contributions presented in the study are included in the article/supplementary material. Further inquiries can be directed to the corresponding author.

## Ethics Statement

The animal study was reviewed and approved by The First Affiliated Hospital of Harbin Medical University.

## Author Contributions

YL and D-WW participated in the research design. YL wrote or contributed to the writing of the manuscript. DW performed the western blotting. B-HD performed the real-time PCR. DW performed immunofluorescence. H-YK performed histopathology and Oil Red O staining. B-HD performed electron microscopy. All authors contributed to the article and approved the submitted version.

## Funding

This work was supported by the National Natural Science Foundation of China (grant no. 81600492).

## Conflict of Interest

The authors declare that the research was conducted in the absence of any commercial or financial relationships that could be construed as a potential conflict of interest.

## Abbreviations

GSDMD, gasdermin D; NASH, non-alcoholic steatohepatitis; NAFLD, non-alcoholic fatty liver disease; NLRP3, nucleotide-binding oligomerization domain-like receptor protein 3; GLP-1, glucagon-like polypeptide-1.
